# Complementary hybrid electrodes for high contrast electrochromic devices with fast response

**DOI:** 10.1038/s41467-019-12617-4

**Published:** 2019-10-25

**Authors:** Carsten Kortz, Alexander Hein, Marius Ciobanu, Lorenz Walder, Egbert Oesterschulze

**Affiliations:** 1 Technische Universität Kaiserslautern, Erwin-Schrödinger-St. 46, 67663 Kaiserslautern, Germany; 2 University of Osnabrück, Barbarastrasse 7, 49074 Osnabrück, Germany

**Keywords:** Applied optics, Optical materials and structures, Optical physics

## Abstract

Fast switching ‘transparent-to-black’ electrochromic devices are currently under investigation as potential candidates in modern applications like e-papers or with additional functionality as ultracompact iris or switchable neutral filter in camera systems. However, recent electrochromic devices show either a lack of contrast or slow response times. To overcome these deficiencies we focus on a careful material composition of the colouring hybrid electrodes in our device. We have established a nanoporous Sb-doped SnO${}_{2}$ electrode as supporting electrode for chemisorbed electrochromic tetraphenylbenzidine molecules due to its good conductivity in the redox potential range of the molecule. This hybrid electrode was combined with a modified nanoporous TiO${}_{2}$ / viologen electrode to realize a high performance, complementary electrochromic device. Fast switching time constants of 0.5 s and concurrently high change in optical density $\Delta$OD = 2.04 at 605 nm confirm our successful concept. The achieved colouration efficiency of 440 cm${}^{2}$ C${}^{-1}$ exceeds every high contrast device presented so far.

## Abstract (web summary)

Electrochromic devices have a range of applications as switchable shutters. Here the authors report a hybrid device that uses complementary electrochromic molecules immobilized on nanoporous electrodes to concurrently achieve fast colouration and bleaching with high contrast over a broad spectral range.

## Introduction

Electrochromic (EC) materials change their spectral transmission when an electric voltage is applied. This reversible phenomenon was first investigated on transition-metal oxides like WO_3_, and later V${}_{2}$O${}_{5}$ and NiO films in the mid-1960s^1–3^. The colouration efficiency (CE) of full cells was substantially improved by introducing prussian blue as the electrochemical complementary material on the counter electrode in WO${}_{3}$ EC devices (ECD) superimposing their spectral absorbance^2^. Although inorganic EC materials benefit from their high chemical stability, the introduction of organic EC materials like derivatives of PEDOT, viologens or arylamines allowed to tune the spectral absorption over a broad spectral range by tailoring their molecular structure^4–8^. All these materials have their low operation voltages accompanied by low- power consumption in common. This enables their use in smart windows, rear-view car mirrors and all transparent-to-coloured displays^9–11^.

The potential of ECDs with their low-power consumption has recently attracted attention for microoptical applications as optical shutters and aperture stops, in particular in smartphone and tablet cameras^12–19^. However, these applications concurrently demand neutral absorption with high contrast and switching times below 1 s, which no ECD presented in the literature could provide yet. In a first attempt to meet these requirements, the above-mentioned concept of electrochemical complementary EC materials was revisited by choosing organic EC materials with additionally complementary optical absorption^10,12,16,20,21^. A controlled variation of the optical density $\Delta$OD = 1.68 @ 600 nm was demonstrated by Deutschmann et al.^16^. However, the use of compact EC material layers as well as EC molecules solved in the electrolyte is hampered by their exceedingly long switching times of several seconds or even tens of seconds due to diffusion processes, limited ion mobility and restricted charge transport through the EC material during the switching process^10,16^.

In more sophisticated ECDs, the electrode set-up was adapted in such a way that a monolayer of EC material is chemisorbed onto mesoporous transparent conductive electrodes to overcome these limitations. Thus, charge transfer is substantially improved by exploiting the conductivity of the mesoporous electrodes, whereas the ion transport takes place in the electrolyte within the nanopores. One of the most convincing EC electrodes of this kind is a hybrid electrode of sintered TiO${}_{2}$ nanoparticles functionalized with an organic monolayer of phosphonated viologen^22–24^. Although excellent switching times of 0.3 s (66% of $\Delta$*T*, active area 1 cm${}^{2}$) were reported, colouration was still restricted to $\Delta$OD = 0.4 @ 608 nm due to the absence of a suitable complementary counter electrode^25^. Approaches by using complementary EC molecules immobilized on mesoporous TiO${}_{2}$ on both electrodes where accomplished by Seong et al.^21^. Although their device reached a rather high colouring efficiency of 250 cm^2^ C^−1^ @ 518 nm the colouration time was always over 2 s.

In this paper, we pursue the successful concept of immobilized electrochemical complementary molecules on sintered nanoparticle layer (NPL) electrodes. However, the concurrent achievement of maximal optical contrast and CE, as well as fast switching capability, requires to fulfill two premises: first it demands a careful material composition of both mesoporous electrodes and the choice of electrochemically as well as spectrally complementary organic EC materials. Each NPL had to provide a high transparent, inert, porous layer with high conductivity in the operating voltage range of the corresponding EC molecule. Second, we had to adapt the cell set-up—in particular the thickness of each NPL electrode as will be discussed below—in such a way that anodic and cathodic coulometry are identical for the desired redox states.

## Results

### Material composition

**Fig. 1:**
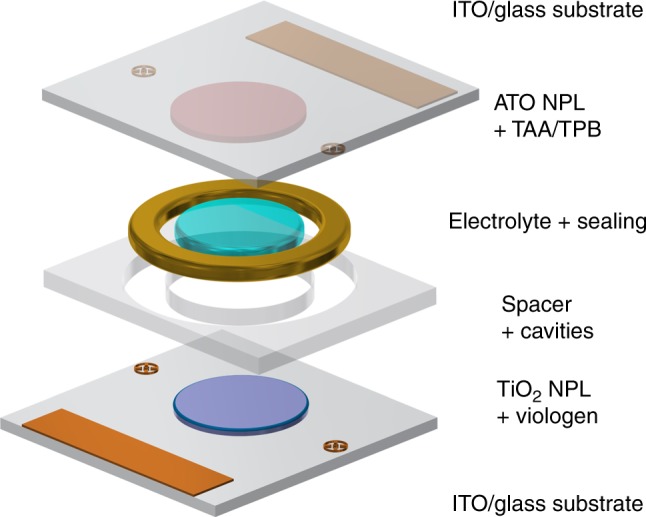
Exploded device scheme of our two-electrode set-up. The scheme shows the cathodic electrode on the bottom, the spacer made of a structured Ordyl photoresist forming the individual cavities for the electrolyte and the UV-curing adhesive as sealing, and the anodic electrode at the top

In accordance with the first premise, our ECD set-up in Fig. 1 relies on two organic complementary EC materials, each chemisorbed onto a NPL electrode, chosen with regard to their electronic behaviour. Prior to the discussion of device response, we will first study each colouring electrode individually, by using a three-electrode set-up.

In the case of the cathodic colouring electrode, we followed the very successful approach of the hybrid electrode consisting of viologen molecules chemisorbed via a phosphonate anchor group onto a TiO${}_{2}$ NPL (particle diameter: 15–20 nm)^24^. The good conductivity of the TiO${}_{2}$ layer in the negative potential range from –0.7 to 0 V, matches the redox potential of the viologen molecule. We found in addition that fast switching of the cathodic electrode also demands a careful thermal treatment of the TiO${}_{2}$ NPL prior to the chemisorption of the viologen molecules. The NPL was built up onto ITO-coated glass substrates by stencil printing and subsequent calcination and sintering at 450°C for 2 h. This heating step was performed in a vacuum furnace at a pressure of 5 mbar to avoid both degradation of the ITO layer and reduction of the TiO${}_{2}$ NPL from titanium-(IV)-oxide to titanium-(III)-oxide, which would lead to a loss of conductivity and transparency, respectively^26–28^.

**Fig. 2:**
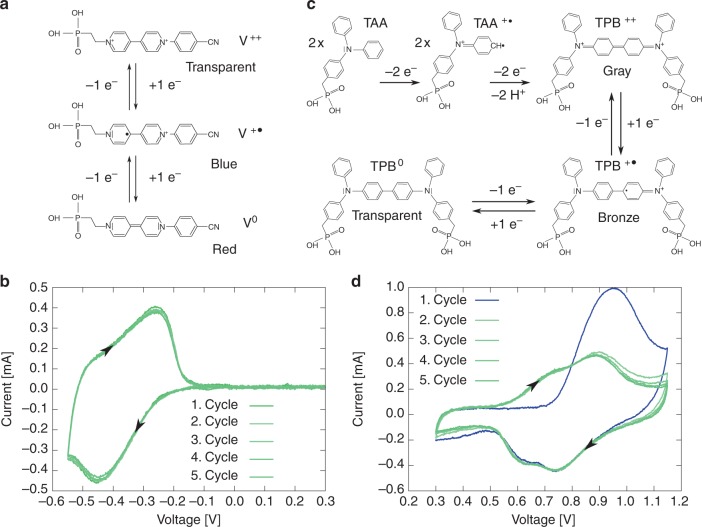
Structural formula of the electrochromic molecules and the corresponding CV measurements. **a** Structural formula of the viologen derivative in the different redox states and **b** the CV measurement in the three-electrode set-up by using an Ag/AgCl reference electrode and Pt counter electrode. **c** Structural formula of the phosphonated TAA and the reaction cascade for TPB formation and **d** CV measurements showing the dimer formation from TAA to TPB also in the above-described three- electrode set-up. Source data are provided as a Source Data file

We chose an asymmetrically substituted viologen bearing a phosphonate acid as anchor group for TiO${}_{2}$ surface and a cyanophenyl group (see Fig. 2a) for enhancement of optical properties in the blue spectral range (compared with previous work^24^). The functionalized TiO${}_{2}$ electrode was cycled from 0.3 to –0.55 V with a scan rate of 10 mV s^−1^ as shown in Fig. 2b to investigate the redox potential of the modified electrode in a three-electrode set-up by using an Ag/AgCl reference electrode and a Pt counter electrode in a propylene carbonate solution containing 1 mol l^−1^ LiClO${}_{4}$. The cathodic current starts at −0.15 V and reaches its maximal value at −0.45 V. The first reduction in the coloured V${}^{+\bullet }$ was completed at −0.55 V yielding a dark-blue colour of the EC film. Further reduction at potentials lower than −0.55 V leads to the neutral state V${}^{0}$ with reddish colour (the spectra of the three redox states are attached in Supplementary Fig. 1 and discussed in more detail in Supplementary Note 1). Because of the chemical instability of the neutral state, no CV measurements were performed at such negative potentials. This also demands to take precautions in the design of our ECD set-up to strictly avoid this state during operation as will be discussed below.

For the best approach of a “transparent-to-black” ECD, we had to adapt the material composition of the complete anodic colouring electrode. The EC material has to be chosen to have an optically as well as electrochemically complementary behaviour with respect to our viologen–TiO${}_{2}$ EC layer. Therefore, we selected tetra-N-phenyl-benzidine (TPB) available by oxidative dimerization of triarylamine (TAA) as the appropriate complementary EC molecule. The use of a TiO${}_{2}$ NPL electrode as support for anodic colouring EC materials proved unsuitable due to its poor conductivity in the positive potential range. Instead, we propose Sb-doped SnO${}_{2}$ (ATO) as an ideal nanoparticle material to establish a fast charge transfer between the TPB and the underlying ITO substrate. So far, ATO NPLs were used solely as a passive ion-storage layer^29^. The preparation of the ATO NPL (particle diameter: 13–22 nm) was performed by using a commercially available ATO nanopowder following the process developed by Cho et al.^30^.

The immobilization of TPB on the ATO NPL electrode was initialized by chemisorption of TAA molecules via a phosphonate acid as anchor group (Fig. 2c). When cycling the electrode from 0.3 to 1.15 V with 10 mV s^−1^ in the above-described three-electrode set-up, it is striking that the first cycle of the anodic electrode differs substantially from the following cycles, which is due to the TPB dimer formation by oxidative coupling of immobilized TAA molecules (Fig. 2d). The following cycles show a highly reversible switching of the anodic electrode. Mechanistic details on the oxidative coupling reaction are given in a separate publication. The current in the CV measurement starts rising at 0.5 V, indicating the oxidation to the radical cationic state (TPB${}^{+\bullet }$) with bronze colour. Further increase in the potential (higher than +0.8 V) leads to the desired dicationic state (TPB${}^{++}$). This reaction is completed at 1.1 V, and the layer shows its pronounced absorption in the red spectral range, which is almost complementary to the absorption of our viologen (see Supplementary Fig. 2 and Supplementary Note 2).

For the discussion of the second premise mentioned above, we need to consider the redox states of the EC materials (see Fig. 2a, c) in our ECD. The optimum spectral response of the chosen complementary EC molecules is achieved if we can concurrently support the colouring redox reactions TPB$${}^{0}$$$$\to$$ TPB$${}^{++}$$ (oxidation potential +1.1 V) and V$${}^{++}\to$$ V$${}^{+\bullet }$$ (reduction potential −0.5 V), avoiding in particular the irreversible V$${}^{0}$$ state. However, when applying an external voltage to our two-electrode ECD, the individual voltage drop at each electrode is a priori not known. The latter is defined by the balance of the Faradaic currents on the respective electrode electrolyte interface. For the desirable colouring reactions, this current balance is achieved if the oxidation of one TBP molecule is accompanied by the reduction of two viologen molecules. This and the protection of viologen towards over-reduction demands the adaptation of the thickness ratio ($${d}_{{{\rm{TiO}}}_{2}}/{d}_{{\rm{ATO}}}$$) of the two NPL layers such that the amount of viologen molecules on the TiO$${}_{2}$$ layer is at least twice the amount of TPB molecules on the ATO layer.

The thickness of the ATO layer was adjusted for a given TiO${}_{2}$ layer of sufficient absorbance (${d}_{{{\rm{TiO}}}_{2}}=3.8$ µm) in such a way that the achieved contrast of the ECD was maximal, and the irreversible V0 state was not observed in the optical spectra. In this way, we fulfilled the condition that the number of viologen molecules was approximately twice the number of TPB molecules for a ATO NPL thickness of ${d}_{{\rm{ATO}}}=5.3$ µm. This was proven by spectroelectrochemical investigations as discussed in the following section.

### Electro-optical characterization

**Fig. 3:**
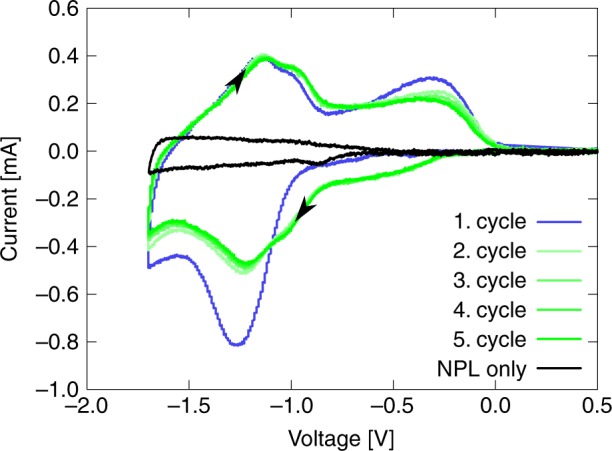
CV measurement of the ECD cycling the device from 0.5 to –1.7 V with a scan rate of 50 mV s^−1^ in the two-electrode configuration, and CV measurement of the bare nanoparticle layers without EC molecules. Source data are provided as a Source Data file

The electrochemical behaviour of our two-electrode ECD was investigated by cyclic voltammetry (CV) by using the anodic electrode as counter and reference electrode simultaneously. We used 1 mol l^−1^ LiClO${}_{4}$ in propylene carbonate as the electrolyte. The voltage was cycled from +0.5 to –1.7 V with a scan rate of 50 mV s^−1^ as shown in Fig. 3. In the first step, we have prepared a device with bare NPLs to identify their intrinsic electrochemical response. The enclosed area of the corresponding CV (black line) in Fig. 3 is small compared with those of ECDs with chemisorbed molecules on the NPLs. No redox peaks are observed, which indicates that no electrochemical side reactions like Li^+^ ion intercalation into TiO${}_{2}$^31^ and no electron and Li^+^ intercalation into ATO occur in the desired potential range^32^. This proves the electrochemical inert electrode behaviour of the bare NPLs, as well as the domination of the faradaic currents in our ECD with the EC molecules attached to the NPLs as claimed above.

As expected from the discussion of TPB, the first cycle in the CV of the ECD also differs from the following cycles due to the TPB formation. All following cycles show a highly reversible switching of the device, which confirms that the TPB formation was almost completed after the first cycle.

In the following cycles, the current starts dropping slowly at a voltage of –0.4 V, indicating the first oxidation state of TPB, and decreases faster at –0.9 V reaching its second oxidation level (compare Fig. 2c). The reduction in viologen at the TiO${}_{2}$ electrode occurs concurrently to the oxidation of TPB and is not distinguishable in the CV. The current becomes maximal at –1.3 V, and the colouration is completed at –1.6 V. To distinguish the two TPB states spectral measurements were conducted.

**Fig. 4:**
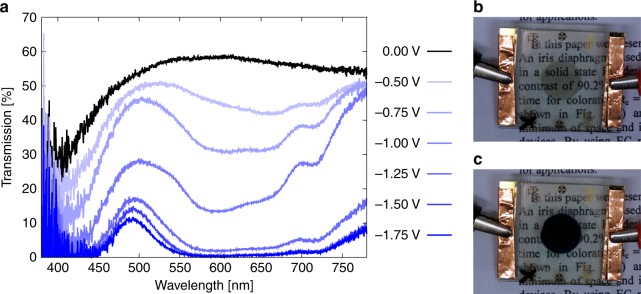
Spectral transmission and images of the device at different voltages. **a** The spectral transmission was recorded from 380 to 780 nm, and images of the device in (**b**): the bleached state at 0 V and **c** coloured state at –1.5 V. The circular switchable area has a diameter of 11 mm. Source data are provided as a Source Data file

The spectral measurements were all taken in the steady state of colouration. Figure 4 shows the transmission through the device for various applied potentials. When a step potential of –1.5 V was applied, the transmission at 605 nm decreased below 0.5%. Thus, we achieved a change in transmission $\Delta$*T* = 57.82% at 605 nm between the transparent state (58.35% at 605 nm) and the coloured state. The corresponding $\Delta$OD at the voltage $V$ can be determined by

1 $$\Delta {{\rm{OD}}}_{{\rm{V}}}({\lambda }_{\max })=\mathrm{log}\frac{{T}_{{\rm{b}}}({\lambda }_{\max })}{{T}_{{\rm{c}}}({\lambda }_{\max })},$$

where ${T}_{{\rm{b}}}({\lambda }_{\max })$ and ${T}_{{\rm{c}}}({\lambda }_{\max })$ is the transmission at the wavelength of maximal transmission change ${\lambda }_{\max }$ in the bleached and coloured state, respectively. This leads to an excellent value of $\Delta$OD${}_{-1.5V}$ (605 nm) = 2.04, which exceeds all high-contrast ECDs in Table 1.

**Table 1:** Comparison of different ECDs

$\Delta$OD (@${\lambda }_{\max }$)	${t}_{c/b}$ [s] for	$x$	$\tau$ [s]	CE [cm^2^ C^−1^]	
2.04 (605 nm)	0.5/0.6	0.9	0.24	440	This paper
0.67 (630 nm)	10/2	0.8	3.7	No data	^15^
1.7 (600 nm)	10/70	0.9	17.3	No data	^16^
0.7 (633 nm)	2/1	0.8	2.0	17	^18^
1.9	5/15	0.9	4.3	290	^20^
1.4	1/2	0.8	0.9	240	^21^
0.47 (608 nm)	0.17/0.42	0.66	0.28	196	^25^
0.5 (650 nm)	2/1	0.9	0.65	18	^32^
0.8 (580 nm)	0.8	0.95	0.27	691	^34^
0.89 (430 nm)	11/81	0.9	20	185	^12^
1.18 (600 nm)	11/15	0.9	5.6	243	^4^
1.31 (550 nm)	0.1/1.1	0.8	0.37	No data	^22^
1.07 (562 nm)	5/10	0.9	3.3	246	^5^

The CIELAB values of the device in the transparent (0.0 V) and coloured state (−1.5 V) were calculated from the respective transmission spectra. For the transparent state we received ${L}_{t}=80.43$; ${a}_{t}=-0.46$; ${b}_{t}=12.02$. The deviation from the neutral colour is caused by the absorption of the NPLs in the blue and UV range. In the coloured state we obtained ${L}_{c}=17.72$; ${a}_{c}=-35.10$; ${b}_{c}=-17.70$. The reduced absorption at 500 nm with its considerable impact on the ${a}_{c}$ and ${b}_{c}$ values is attributed mainly to the material properties of viologen (see Supplementary Fig. 1). Nevertheless, the variation $\Delta L=62,71$ obtained by switching between the transparent and coloured state is promising for practical applications.

As can also be seen in Fig. 4, the transmission of the device can not only be switched from *T*${}_{\max }$ to *T*${}_{\min }$, but also to any desired intermediate value. For low voltages, the transmission spectra are characteristic for the first radical cationic V${}^{+\bullet }$ state of viologen ^24^ (see Supplementary Fig. 1), indicated by a strong absorption from 550 to 650 nm and 400 to 450 nm with a specifically higher transmission at 780 nm. The spectral data also prove that the V${}^{0}$ state was successfully avoided in the operating voltage range.

The different oxidation states of TPB have a noticeable effect on the spectra in Fig. 4. The oxidation to the first radical cationic TPB${}^{+\bullet }$ state (–0.5 to −1 V) appears bronze, which also leads to a high transmission in the wavelength range of 700–780 nm. Further oxidation to the dicationic TPB${}^{++}$ is indicated by the large transmission drop at 780 nm, which can be observed at a voltage of ca. −1.25 V in our device.

As expected from the spectral characterization of the two materials in the three-electrode set-up, the combination of the V${}^{+\bullet }$ and TPB${}^{++}$ state gives a high and almost neutral absorption. The spectral measurements prove that the desired redox states were realized in the device as claimed above.

Optimum EC switching performance can be granted if the CE is high, as the CE is defined as the change in optical density $\Delta {{\rm{OD}}}_{{\rm{v}}}({\lambda }_{\max })$ per charge density $\sigma$

2 $${\rm{CE}}=\frac{\Delta {{\rm{OD}}}_{{\rm{v}}}({\lambda }_{\max })}{\sigma }.$$

**Fig. 5:**
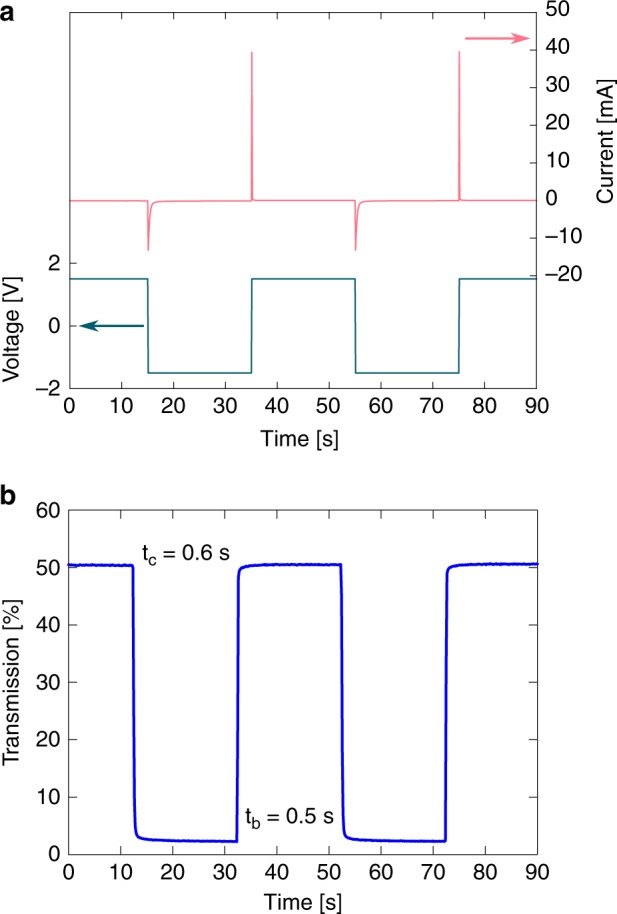
Electro-optical characterization of the electrochromic device. **a** Chronoamperometry measurement by applying a voltage of $\pm$1.5 V for 20 s each and **b** the simultaneously recorded transmission integrated over the visible range (380–780 nm). Source data are provided as a Source Data file

The charge density can be determined by integrating the current from the chronoamperometric (CA) measurements by applying a step potential of $\pm$1.5 V and recording the corresponding current. As can be seen from the CA in Fig. 5a, the current reaches high values of −12 and 40 mA for colouration and bleaching, respectively, and drops rapidly to 0 mA after the change in colouration is performed. The determined charge density was 4.6 mC cm^−2^. This results in an excellent CE of 440 cm^2^ C^−1^, which proves the high suitability of our ECD in battery-powered devices.

## Discussion

In order to investigate the suitability for optical applications, both the optical switching time and contrast over the whole visible range had to be considered. For applications the Michelson contrast defined as

3 $$C\ =\ \frac{{T}_{{\rm{b}}}^{{\rm{int}}}-{T}_{{\rm{c}}}^{{\rm{int}}}}{{T}_{{\rm{b}}}^{{\rm{int}}}+{T}_{{\rm{c}}}^{{\rm{int}}}},$$

is essential where ${T}_{{\rm{b/c}}}^{{\rm{int}}}$ are the integrated transmission values from 380 to 780 nm in the bleached and coloured state, respectively.

CA curves and the corresponding in situ-integrated transmission from 380 to 780 nm were recorded as response to step potentials of $\pm$1.5 V and are shown in Fig. 5a, b, respectively. The switching time for colouration and bleaching is denoted as ${t}_{{\rm{c}}}$ and ${t}_{{\rm{b}}}$, respectively. It is defined as the time from applying the potential till the time when 90% of the transmission change is completed. After the potential was applied, the transmission dropped rapidly to 2.5% and reached the initial value of 50.5% after the reversed potential was applied. The determined optical Michelson contrast was 90.6% for the whole visible range, and the switching times were 0.6 and 0.5 s for colouration and bleaching, respectively. Data concerning the cyclic stability of the device are provided in Supplementary Fig. 3 and discussed in more detail in Supplementary Note 3. It shows that the contrast after 500 cycles of operation is still 83% of the initial contrast. Even after a storage time of 4 months, a degradation of the ECD properties was not observed. The switching of our device in real time is demonstrated in Supplementary Movie 1.

To allow a comparison of our switching time with those of the different ECDs in the literature, we followed the method of Hassab et al.^33^ by using the equation

4 $$\Delta T(t)=\Delta {T}_{\max }\left(1-\exp \left(-\frac{t}{\tau }\right)\right).$$

The time constant $\tau$ can be calculated from the switching times to an arbitrary fraction $x$ of $\Delta {T}_{\max }$ by using

5 $$\tau =\frac{{t}_{{\rm{c/b}}}(x)}{{\rm{ln}}\left(\frac{1}{1-x}\right)}.$$

For our ECD ${t}_{{\rm{c/b}}}$ was measured at 90% of $\Delta {T}_{\max }$ ($x$ = 0.9) and results in $\tau$ = 0.24 s. We have also evaluated $\tau$ for competing ECDs from the literature and collected these data in Table 1. It shows that our time constant $\tau$ is lower, and we received concurrently the largest reported $\Delta$OD.

The Michelson contrast or the integrated transmission over the whole spectral range were not reported in most references mentioned in Table 1 although these parameters are essential for optical applications. A contrast of 90.6% over the whole visible range along with short switching times (0.5 s) is an important step towards the widespread applications of ECDs.

The concept of electrochemically as well as spectrally complementary EC materials, immobilized on an adapted mesoporous, inert electrode with high conductivity in the desired potential range, was successfully demonstrated. The understanding of the electrochemical reactions on each colouring electrode was important to adapt the device set-up in such a way that only the desired redox states of the complementary EC materials contributed to the colouration of the ECD. The concurrent realization of high $\Delta$OD, low switching time and also high CE values exceeds the performance of most ECDs presented so far in the literature. This is an important step towards the application of these microfabricated, material-adapted ECDs for space-limited optical applications in ‘transparent-to-black’ displays like smartphones, tablets and smart watches.

## Methods

### Device fabrication

The device was built up of two ITO-coated glass sheets (8–12 $\Omega$ sq^−1^, Sigma Aldrich). These substrates were sonicated in acetone, isopropyl alcohol and DI water for 5 min each. Alignment marks and contact pads made of 90-nm Au with a 10-nm Cr adhesion layer were added by using magneton sputtering (Oerlikon UNIVEX 450 C). The mesoporous TiO${}_{2}$ layer was coated onto the cathodic electrode by stencil printing (Mechatronic systems S40) of a TiO${}_{2}$ nanoparticle paste (Solaronix, Ti-Nanoxide T/SP) on a circular area of 1.1 cm in diameter (A${}_{{\rm{EC}}}$ = 0.95 cm^2^) by using a 30 µm-thick metal mask. This paste contains the nanoparticles, organic components and a solvent. The coated samples were heated slowly to 450 °C with a ramp of 10°C min^−1^ in a vacuum furnace (Carbolite Gero GHA 12/1200) at a pressure of 5 mbar, remained under these conditions for 2 h and cooled down naturally. During this process, the solvent evaporated, and the organic components decomposed, leaving a conductive transparent porous layer on whose surface the EC molecules (viologen) were chemisorbed. To provide a maximal surface coverage with EC molecules, the samples were kept in a $5 \,\times \, 1{0}^{-3}\,{\rm{mol}}\ {{\rm{l}}}^{-1}$ solution of viologen in ethanol/H${}_{2}$O 90/10 for 15 h. The anodic electrode was coated with an ATO nanoparticle paste by using commercially available nanopowder (Alfa Aesar, Sb${}_{2}$O${}_{5}$:SnO${}_{2}$, APS 13–22 nm) as a source of ATO nanoparticles. The samples were coated by using the same stencil printer and a 70 µm-thick metal mask and heated to 450 °C under the same conditions as described above. Subsequently, the anodic electrode was kept in a $2 \,\times\, 1{0}^{-3}{\rm{mol}}\ \,{{\rm{l}}}^{-1}$ solution of TAA salt in ethanol under the absence of oxygen for 15 h.

A spacer (Elga Europe, Ordyl SY355) was added to the substrate carrying the anodic electrode. It was structured by using UV lithography. This structured spacer forms the cavities for the electrolyte (1 mol l^−1^ LiClO${}_{4}$ in propylene carbonate) and the UV-curing adhesive (Photowell 1197, Wellmann Technologies GmbH), which encloses the cavity for the electrolyte and provides the hermetic sealing and the stability of the device. To ensure that the EC molecules were not affected by oxygen and moisture, the whole assembly routine was performed under exclusion of O${}_{2}$ and H${}_{2}$O in an Ar glove box (GS Mega). The cavities for electrolyte and adhesive were subsequently filled with 4.2 and 8 mg of electrolyte and UV-curing adhesive, respectively, by using a 3-axes dispenser (Musashi Image Master 350 PC Smart). The two electrodes were aligned, the NPLs facing each other, in a customized aligner with a lateral offset of 5 mm, for electrical contact. The substrates were pressed together with a force of 60 N and illuminated with UV light for 180 s, resulting in a hermetic sealed ECD.

### Measurement set-up

The spectroelectrochemical measurements were performed under ambient conditions. The electrodes of the device were connected to a potentiostat (Gamry Reference 600), and the device was investigated in a two-electrode configuration. The cathodic electrode was operated as the working electrode, whereas the anodic electrode was used as the counter and reference electrode, simultaneously. CV measurements were performed to identify the reduction and oxidation peaks of the material combination used in the device. The potential was cycled from 0.5 to –1.7 V with a scan rate of 50 mV s^−1^. The transmission of the device was characterized by illuminating the whole electrochromic area by a white light source (Schott KL 1500 LCD) and recording the transmitted light by using an integrating sphere and a spectrometer (Ocean Optics, flame). The spectral information of the transmitted light was recorded for different applied potentials (0 to −1.75 V) by using the same device. All spectral measurements were taken in a steady colouration state. For switching time characterization a step potential of $\pm$1.5 V was applied for 20 s each. The transmitted light was integrated from 380 to 780 nm and recorded over time. The switching time (*t*${}_{{\rm{c/b}}}$) was defined as the time when 90% of the transmission change was completed. The change in OD was calculated from the transmission spectra at 605 nm averaging it from 600 to 610 nm. The charge density was calculated by integrating the current from the chronoamperograms for colouration and bleaching.

## Supplementary information

Supplementary Information

Supplementary Movie 1

Source Data

## Data Availability

All data supporting the findings in this study and underlying Figs. 2b, d, 3–5 and Supplementary Figs. 1–3 are provided as a Source Data file.
