# Supplementary material for: Complementary hybrid electrodes for high contrast electrochromic devices with fast response
Source: Nat Commun. 2019 Oct 25;10:4874. doi: 10.1038/s41467-019-12617-4 (PMC6814761; doi:10.1038/s41467-019-12617-4)
Supplement: Supplementary file 1 — Supplementary Information [file 41467_2019_12617_MOESM1_ESM.pdf]

Complementary Hybrid Electrodes for High Contrast Electrochromic Devices with Fast Response

Carsten Kortz, Alexander Hein, Marius Ciobanu, Lorenz Walder, Egbert Oesterschulze

Supplementary Note 1

To identify the spectral response of each EC material used in our devices, we present transmission spectra of viologen (*Fig. S1*) and TPB (*Fig. S2*) separately. The measurements were performed in a three-electrode set-up with the EC electrode as working electrode, an Ag/AgCl reference electrode, and a Pt counter electrode using 1 mol/l $LiClO_4$ solved in propylene carbonate as electrolyte.

The transmission spectra of the cathodic coloring electrode are shown in Supplementary Fig. 1. The observed change in transmission is solely due to the reduction of the viologen molecules.

The dicationic state V^{++} (yellow line) has a high and almost homogeneous transmission in the visible range, whereas the radical cationic state $V^{+\bullet}$ (blue line at -0.5 V) shows the desired transmission for the viologen used in our device. The neutral state V^{0} (red line at -1 V) has a rather low transmission at ca. 500 nm but a very high transmission for wavelengths lager than 600 nm. High voltage drops at the cathodic electrode should be avoided, due to the irreversible nature of the V^0 state.

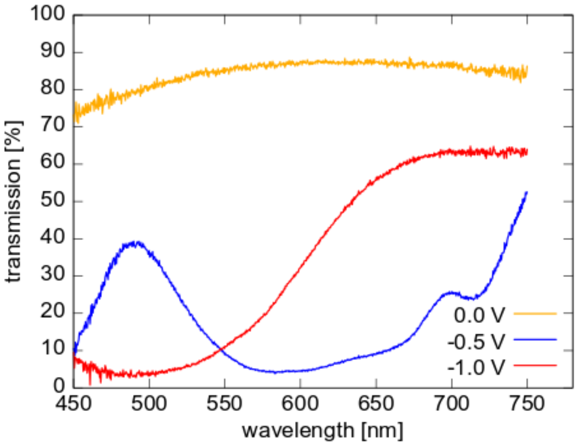

Supplementary Figure S1. Spectral transmission through the viologen / TiO2 electrode in the three different redox states.

Supplementary Note 2

The different oxidation states of the anodic coloring electrode were also investigated in the three-electrode set-up described above and the according transmission spectra are displayed in Supplementary Fig. 2.

The high transmission in the visible range is due to the neutral TPB^0 state (yellow line at 0.0 V). The first oxidized radical cationic state $TPB^{+\bullet}$ (blue line at 0.7 V) shows a rather high transmission for wavelengths larger than 550 nm, whereas the desired dicationic state TPB^{++} (blue line at 1.2 V) has a low transmission for wavelengths larger than 500 nm.

The combination of these complementary materials in a single device gives rise to the high absorption observed in the visible range.

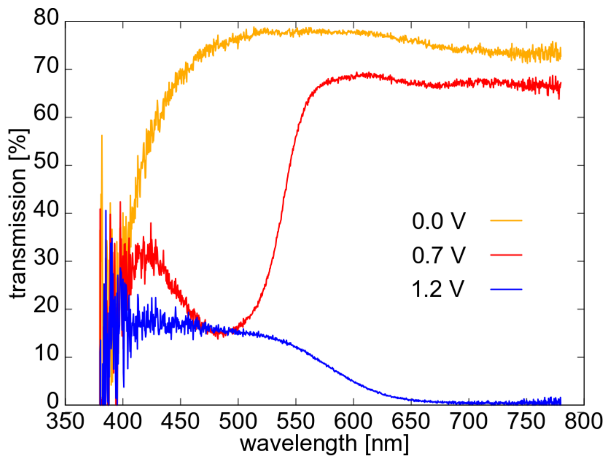

Supplementary Figure S2. Spectral transmission through the TPB / ATO electrode in the different redox states.

Supplementary Note 3

The transmission of the hermetically sealed device measured in the two electrode configuration for 500 cycles is displayed in Supplementary Fig. 3.

For the stability measurements of the ECD's an OLED panel (SeelectorLux, HEMA Electronics, Germany) was used for illumination because of its higher temporal stability over several hours. In the transparent state no degradation of the ECD is visible. After 500 cycles of operation the contrast (ΔT) of the ECD is still 83 % of the initial value. The data in *Fig. S3* were recorded with the same device after accomplishing the extensive characterization and imaging processes mentioned in the manuscript.

Although some electrochromic polymer materials (in particular PEDOT based derivatives) show a higher stability, none of them reaches concurrently high Michelson contrast and low switching time over the entire optical range.

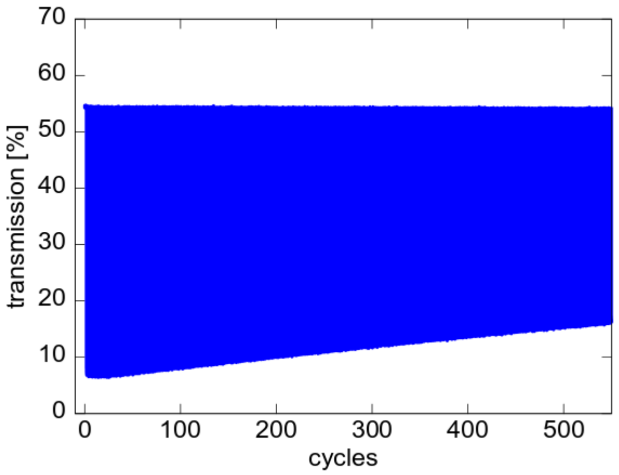

Supplementary Figure S3. Integrated transmission (380 – 780 nm) through the device applying a square wave voltage of ±1.5 V for 500 cycles with a duty cycle of 50% and a period of 40 s.
